# Enzyme-Based Solid-Phase Electrochemiluminescence Sensors with Stable, Anchored Emitters for Sensitive Glucose Detection

**DOI:** 10.3390/bios15050332

**Published:** 2025-05-21

**Authors:** Chunyin Wei, Yanyan Zheng, Fei Yan, Lifang Xu

**Affiliations:** 1 Guangxi Medical University Cancer Hospital, Guangxi Medical University, Nanning 530021, China; chunyinwei@sr.gxmu.edu.cn; 2 Postdoctoral Research Center, School of Basic Medicine, Guangxi Medical University, Nanning 530021, China; 3 School of Chemistry and Chemical Engineering, Zhejiang Sci-Tech University, Hangzhou 310018, China; 201920103007@mails.zstu.edu.cn; 4 The Second Affiliated Hospital of Guangxi Medical University, Nanning 530007, China

**Keywords:** enzyme sensor, electrochemiluminescence, immobilized emitter, nanochannel array, glucose

## Abstract

Glucose (Glu) detection, as a fundamental analytical technique, has applications in medical diagnostics, clinical testing, bioanalysis and environmental monitoring. In this work, a solid-phase electrochemiluminescence (ECL) enzyme sensor was developed by immobilizing the ECL emitter in a stable manner within bipolar silica nanochannel array film (bp-SNA), enabling sensitive glucose detection. The sensor was constructed using an electrochemical-assisted self-assembly (EASA) method with various siloxane precursors to quickly modify the surface of indium tin oxide (ITO) electrodes with a bilayer SNA of different charge properties. The inner layer, including negatively charged SNA (n-SNA), attracted the positively charged ECL emitter tris(2,2′-bipyridyl)ruthenium(II) (Ru(bpy)_3_^2+^) via electrostatic interaction, while the outer layer, including positively charged SNA (p-SNA), repelled it, forming a barrier that efficiently concentrated the Ru(bpy)_3_^2+^ emitter in a stable manner. After modifying the amine groups on the p-SNA surface with aldehyde groups, glucose oxidase (GOx) was covalently immobilized, forming the enzyme electrode. In the presence of glucose, GOx catalyzed the conversion of glucose to hydrogen peroxide (H_2_O_2_), which acted as a quencher for the Ru(bpy)_3_^2+^/triethanolamine (TPA) system, reducing the ECL signal and enabling quantitative glucose analysis. The sensor exhibited a wide linear range from 10 μM to 7.0 mM and a limit of detection (LOD) of 1 μM (S/N = 3). Glucose detection in fetal bovine serum was realized. By replacing the enzyme type on the electrode surface, this sensing strategy holds the potential to provide a universal platform for the detection of different metabolites.

## 1. Introduction

Glucose (Glu) detection, a fundamental analytical technique, has critical applications in various fields [1]. In medical diagnostics, blood glucose monitoring is vital for managing metabolic diseases like diabetes [2,3]. The World Health Organization (WHO) reports that the global incidence of diabetes continues to rise. From 1990 to 2022, the prevalence of diabetes among adults worldwide increased from 7% to 14%, with the number of patients increasing more than four-fold and now exceeding 800 million [4]. The International Diabetes Federation (IDF) has released the 11th edition of the IDF Diabetes Atlas. In 2024, approximately 3.4 million people worldwide were expected to die from diabetes or its complications, accounting for 9.3% of all-cause mortality among adults aged 20–79 [5]. By tracking blood glucose concentrations, it provides key parameters for disease screening, treatment adjustments, and complication prevention. In clinical testing, glucose detection is used not only for routine biochemical analysis but also for precision diagnostics, such as identifying tumor metabolic characteristics. In the food industry, this technology serves dual purposes for quality control. It ensures the stability of product flavor and texture by monitoring glucose levels in raw materials and finished products while also serving as a legal test for food additive management and nutrition label verification [6]. In biomanufacturing, glucose, the primary carbon source for microbial fermentation, significantly impacts the efficiency and purity of bio-products like antibiotics and enzymes. Its concentration detection is a critical control point for optimizing fermentation. In environmental monitoring, determining glucose levels in water bodies helps assess organic pollution levels, providing scientific support for wastewater treatment and ecosystem health evaluation [7]. Thus, sensitive and convenient glucose detection technology is crucial for maintaining health, optimizing industrial production, and protecting the environment.

Current glucose detection methods primarily include electrochemical and optical techniques. Electrochemical methods primarily involve enzyme electrode methods and non-enzyme catalytic methods [8,9]. The former relies on glucose oxidase (GOD) for specific recognition, while the latter uses nanomaterials to directly catalyze the oxidation of glucose. However, the stability and reproducibility of electrodes still require improvement. Optical methods encompass fluorescence probes, colorimetric sensing, or surface plasmon resonance technologies, offering the advantage of visual detection, but they are prone to interference from sample matrices [10,11,12]. Among these methods, electrochemical and fluorescent methods typically exhibit lower detection limits (commonly 1 μM~10 μM), whereas colorimetric and SPR techniques generally have higher detection limits (commonly 10 μM~100 μM) [13,14,15]. The improvement in detection limits is often based on signal amplification by functionalized nanomaterials [16,17]. Electrochemiluminescence (ECL) is an analytical method that combines electrochemical and chemiluminescent reactions [18,19,20]. It generates light signals through electrochemical reactions on the electrode surface. Its unique advantages include the absence of an external light source, the ease of miniaturization and integration, low background noise, a high signal-to-noise ratio, and low detection limits (commonly 0.1 μM~1 μM for glucose detection) [21,22,23,24]. In the solid-state ECL mode, in particular, where the ECL emitter is anchored to the electrode surface, the ECL signal is stable [25,26]. This design also allows for the reduction in probe usage through suitable enrichment effects. The products generated by enzymatic reactions in situ serve as co-reactants or quenchers for ECL, providing a highly efficient and specific new strategy for biomolecular detection [27]. For instance, GOD catalyzes the conversion of glucose to hydrogen peroxide (H_2_O_2_). The generated H_2_O_2_ acts as a quencher for ECL emitters such as tris(bipyridyl)ruthenium (Ru(bpy)_3_^2+^), reducing the ECL signal. This process not only directly associates target glucose with the ECL signal but also significantly improves detection specificity due to the high selectivity of the enzyme [28,29]. Thus, this design combines the biomolecular recognition function of the enzymatic reaction with the high sensitivity of ECL, providing a new approach for selective and highly sensitive glucose detection.

In recent years, a variety of approaches have been investigated to address the specific challenge of glucose sensing. Firstly, nanomaterial-based strategies have been employed, incorporating nanomaterials that selectively bind to glucose, thereby minimizing interference from other biomolecules [30]. Secondly, surface functionalization techniques have been developed, where the sensor surface is functionalized with glucose-specific recognition elements, such as glucose-binding proteins, antibodies, or aptamers, to enhance specificity [31]. Thirdly, enzyme-based glucose sensors, particularly those utilizing glucose oxidase (GOx), have demonstrated high sensitivity and selectivity for glucose [32]. In such enzyme-catalyzed detection systems, the use of free or immobilized enzymes directly affects detection performance [33,34]. Free enzymes participate directly in liquid-phase catalytic reactions, rapidly generating products, but they suffer from instability, irreversible deactivation, and the inability to be reused. Moreover, the random diffusion of enzymatic products leads to low local concentrations at the electrode interface, which affects detection sensitivity and signal stability. In contrast, immobilized enzymes, fixed via physical adsorption, encapsulation, or chemical bonding, significantly enhance the enzyme’s conformational stability and operational durability. Additionally, the products generated in the enzymatic reaction achieve a high concentration at the electrode interface, improving signal intensity. Immobilized enzyme systems also enable sensor reuse and miniaturized integration, effectively reducing detection costs. Among enzyme immobilization strategies, covalent immobilization offers notable advantages over physical adsorption or encapsulation. By chemically anchoring GOD to the functionalized electrode surface, this approach prevents enzyme detachment due to weak interactions in physical adsorption and maintains the spatial freedom of the active site by modulating the electrode modification layer and maximizing catalytic activity [35,36]. This covalent immobilization strategy has great potential for the development of highly stable and sensitive glucose biosensors.

The introduction of nanomaterials to modify electrodes is a key strategy for optimizing immobilized enzymes and probes [37]. The high surface area of nanomaterials significantly enhances the probe loading density, improves interfacial electron transfer efficiency, and reduces the required number of probes [38,39]. Additionally, the abundant active sites on the surface of nanomaterials can chemically anchor enzymes while also increasing the concentration of enzymatic products at the electrode. Silica nanochannel array films (SNA) offer a unique nanochannel array with excellent permeability [40]. Electrodes modified with SNA exhibit several advantages. First, the highly oriented nanochannel structure provides a well-defined confined space or carrier for the loading of ECL emitters and enzymes. Specifically, its large surface area and abundant silanol groups facilitate salinization coupling reactions for modification, allowing the introduction of enzymes. In addition, the SNA nanochannel wall contains abundant silanol groups (p*K*_a_~2), which, under normal solution conditions, deprotonate to give a negatively charged surface [41,42,43]. This allows for the effective enrichment of positively charged ECL emitters via electrostatic interactions [44,45,46]. Second, the vertically aligned nanochannel array of SNA can synergistically promote mass transport [47,48,49]. For example, when enzymes are immobilized on the outer surface of the SNA and also near the entrance of the nanochannels, the enzymatic reaction products can diffuse to the electrode surface, increasing the concentration on the electrode surface and enhancing detection sensitivity. Third, the size effect of the nanochannels can selectively filter out interfering substances [50,51,52,53]. For instance, the 2–3 nm nanochannels of SNA can exhibit size exclusion effects, blocking particles, organelles, proteins, and other large substances from entering the nanochannels, and thus preventing non-specific adsorption from complex matrices that could avoid electrode contamination [54,55,56]. Therefore, SNA-modified electrodes hold great potential for constructing high-performance enzyme-based solid-phase ECL sensors for glucose detection.

**Figure 1:**
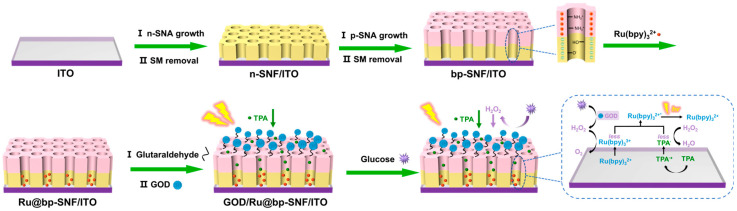
Schematic illustration for the construction process of bipolar bilayer SNA and its application in solid-state ECL enzyme sensors for glucose detection.

In this work, a bilayer SNA with different types of charge was grown on the electrode surface to electrostatically enrich the ECL emitter and immobilize enzymes for sensitive glucose detection. As shown in Figure 1, the bilayer SNA consisted of a dual-layer structure with asymmetric surface charges, also referred to as bipolar SNA (bp-SNA). The inner layer of the SNA carried a negative charge, while the outer layer was positively charged. This electrostatic cage structure is stable and can enrich the cationic ECL emitter Ru(bpy)_3_^2+^. The amino groups on the outer layer of the SNA were derivatized with aldehyde groups and covalently bound to GOD. When glucose was present in the solution, H_2_O_2_ was generated in situ through the enzymatic reaction and acted as a quencher to reduce the ECL signal of the Ru(bpy)_3_^2+^/tri-propylamine (TPA) system. Based on this mechanism, sensitive glucose detection can be achieved.

## 2. Materials and Methods

### 2.1. Chemicals and Materials

The chemical reagents used in this study were all of analytical reagent grade and were used directly without further purification. The water used in the experiments was ultrapure water with a resistivity higher than 18.2 MΩ·cm. The main reagents, including tetraethyl orthosilicate (TEOS, 98% purity), cetyltrimethylammonium bromide (CTAB), potassium ferrocyanide (K_3_[Fe(CN)_6_], 99.5%), potassium ferricyanide (K_4_[Fe(CN)_6_], 99.5%), bovine serum albumin (BSA), (3-aminopropyl)triethoxysilane (APTES), glucose (Glu), glucose oxidase (GOx), and glutaraldehyde (GA), were all purchased from Aladdin Biochemical Technology Co., Ltd. (Shanghai, China). The ECL emitter tris(2,2′-bipyridyl)ruthenium(II) chloride hexahydrate (Ru(bpy)_3_Cl_2_·6H_2_O) and redox probe tris(ammonium)ruthenium(III) chloride (Ru(NH_3_)_6_Cl_3_) were obtained from Sigma-Aldrich (Shanghai, China). Fetal bovine serum (FBS) was purchased from Macklin Biotech Co., Ltd. (Shanghai, China). Indium tin oxide (ITO) conductive glass was purchased from Kaiwei Optoelectronics Technology Co., Ltd. (Zhuhai, China).

### 2.2. Measurements and Instrumentations

The thickness analysis of the bp-SNA samples was conducted using a SU8010 scanning electron microscope (SEM, Hitachi, Tokyo, Japan, under an accelerating voltage of 5 kV) and a transmission electron microscope (TEM, HT7700, Tokyo, Japan, under an accelerating voltage of 100 kV). Electrochemical (EC) analysis was performed using a Metrohm Autolab PGSTAT302N electrochemical workstation (Metrohm, Herisau, Switzerland), which included three modes: electrochemical impedance spectroscopy (EIS), cyclic voltammetry (CV), and differential pulse voltammetry (DPV). The DPV test parameters were set as follows: a potential step size of 5 mV, pulse amplitude of 50 mV, pulse duration of 50 ms, and sampling interval of 200 ms. Electrochemiluminescence (ECL) signal collection was achieved using the Xi’an RuiMai MPI-E II analytical system. In the case of EC and ECL analysis, the tests were carried out in a standard three-electrode system at room temperature. Briefly, the Ag/AgCl electrode was used as the reference electrode, the platinum wire electrode acted as the counter electrode, and bare ITO or its functionalized modified electrode (effective area 0.5 cm^2^) was the working electrode.

### 2.3. Growth of Bilayer SNA on the Electrode Surface

The bilayer SNA was grown on cleaned ITO glass using an electrochemical-assisted self-assembly method (EASA) [57,58,59]. Before use, the ITO glass was sonicated so that it could be cleaned in a 1 mol/L NaOH solution for 1 h, followed by sequential ultrasonic cleaning with acetone, ethanol, and ultrapure water for 15 min, respectively. The SNA preparation steps were as follows: 20 mL of ethanol, 20 mL of NaNO_3_ (0.1 M, pH 2.6), 1.585 g of CTAB, and 2.833 g of TEOS were mixed and stirred at room temperature for 2.5 h to obtain the precursor solution. Using a three-electrode system, the ITO electrode served as the working electrode. The bilayer SNA grew with a current density of −1.3 mA/cm^2^ for 10 s. The electrode was quickly removed for thorough washing. It was then aged overnight at 120 °C to obtain the surfactant micelle-containing SM@n-SNA/ITO electrode. The SM@n-SNA/ITO electrode was immersed in a 0.1 M HCl–ethanol solution and stirred for 5 min to remove the micelles, resulting in a nanochannel-opening n-SNA/ITO electrode.

Next, the second layer of amine-modified SNA was grown. The n-SNA/ITO electrode was placed in a precursor solution containing APTES. Specifically, 1 mM APTES was added to the precursor solution. The second SNA layer (p-SNA) was grown using the same constant current method for 10 s, followed by rinsing and aging, yielding the bp-SNA-modified electrode with SM (SM@bp-SNA/ITO). Finally, the SM was removed in the HCl–ethanol solution, and an electrostatic cage-modified electrode (bp-SNA/ITO) was obtained.

### 2.4. Preparation of Enzyme Electrode and Immobilization of ECL Emitter

The enzyme electrode was prepared using a glutaraldehyde crosslinking method [60]. Specifically, the bp-SNA electrode was then immersed in a 1% GA solution and reacted at 37 °C in the dark for 30 min. The electrode was washed with 0.01 M PBS (pH 7.4) to remove unbound GA, yielding the aldehyde-modified electrode, referred to as the GA/bp-SNA/ITO electrode. The GA/bp-SNA/ITO electrode was then immersed in a 10 mM Ru(bpy)_3_^2+^ solution and stirred for 1 h to enrich the ECL emitter, followed by washing with 0.01 M PBS to remove any unpenetrated Ru(bpy)_3_^2+^, resulting in an electrode with an immobilized ECL emitter (Ru@GA/bp-SNA/ITO). Finally, the Ru@GA/bp-SNA/ITO electrode was immersed in a GOx solution and incubated overnight at 4 °C for Gox-attaching. After washing with PBS (0.01 M, pH 7.4) to remove unbound GOx, the GOx/Ru@GA/bp-SNA electrode was obtained.

### 2.5. Detection of Glucose

The electrolyte solution used was 0.01 M PBS (pH = 7.4), containing 3 mM TPA. After the GOx/Ru@GA/bp-SNA electrode was exposed to different concentrations of standard glucose solution, the ECL signal of the electrode was measured. The ECL intensity (*I*) was fitted with a glucose concentration (*C*_glucose_) to obtain a standard linear response for glucose detection. The linear range for glucose detection was determined based on the glucose concentration range in the linear regression curve. The LOD was calculated based on a signal-to-noise ratio of 3 (S/N = 3), and the limit of quantification (LOQ) was calculated based on a signal-to-noise ratio of 10 (S/N = 10). For real sample analysis, the glucose content in FBS was determined using the standard addition method. Specifically, glucose solutions with known concentrations were added to FBS and then diluted 50-fold with 0.01 M PBS (pH 7.4) containing 3 mM TPA. The glucose concentration was determined based on the standard calibration curve. The recovery was calculated by comparing the measured glucose concentration with the spiked standard concentration. The relative standard deviation (RSD) was calculated based on three replicate measurements.

## 3. Results and Discussion

### 3.1. Preparation of Bipolar Bilayer SNA and Characterization of bp-SNA-Modified Electrode

Figure 1 illustrates the construction process of the bipolar bilayer SNA and its application in a solid-state ECL enzyme sensor for glucose detection. In this study, the ITO electrode was employed as the supporting electrode. The ITO electrode exhibited good electrical conductivity, high optical transparency, and chemical stability. Its surface can be readily functionalized, making it highly suitable for the development of diverse sensing platforms [61,62]. Moreover, ITO is cost-effective, can be fabricated into disposable formats, and is well-suited for patterning and miniaturization [63,64]. An electrochemically assisted self-assembly (EASA) method was employed to efficiently construct mesoporous silica nanochannels on the ITO electrode surface. This EASA method provides a controllable approach for synthesizing SNA materials, offering advantages such as a short preparation time and easy operation. Specifically, under the application of appropriate negative potential, the cationic surfactant CTAB forms micellar templates that rapidly self-assemble on the electrode surface through electrostatic interactions. During the electrolysis process, the reduction in water molecules and H^+^ led to a significant increase in the OH- concentration at the interface, triggering the condensation reaction of the siloxane precursor. Due to the electrostatic attraction between the cetylammonium (CTA^+^) cations and the negatively charged silica groups, a highly ordered hexagonal mesostructure can form rapidly within seconds under kinetic control.

For the growth of the bipolar SNA, TEOS was used as a precursor to prepare the negatively charged inner n-SNA layer, followed by the introduction of APTES to construct the positively charged outer p-SNA layer, resulting in the final bipolar bilayer structure-modified electrode (bp-SNA/ITO). After surface amination treatment, the outer layer formed active aldehyde groups at the interface, enabling the covalent immobilization of GOx molecules. Notably, under stirring conditions, the cationic ECL emitter Ru(bpy)_3_^2+^ could overcome the electrostatic repulsion of the p-SNA layer and be selectively enriched by the inner n-SNA layer. This unique bipolar electrostatic environment stabilized Ru(bpy)_3_^2+^ within the nanocavities. In brief, the negatively charged inner layer generated strong electrostatic adsorption, while the positively charged outer layer created an effective electrostatic barrier. When glucose was introduced into the system, GOx catalyzed the oxidation of the substrate to generate H_2_O_2_. This product quenched the ECL signal of the Ru(bpy)_3_^2+^-TPA system, establishing a quantitative relationship between ECL intensity and glucose concentration.

Thus, the bipolar SNA structure enabled the stable immobilization of the ECL emitter, while surface aldehyde modification allowed the directional immobilization of enzyme molecules. This design strategy effectively integrated the specificity of enzyme-catalyzed reactions with the high sensitivity characteristics of solid-phase ECL detection, providing a new approach for the development of glucose biosensors.

**Figure 2:**
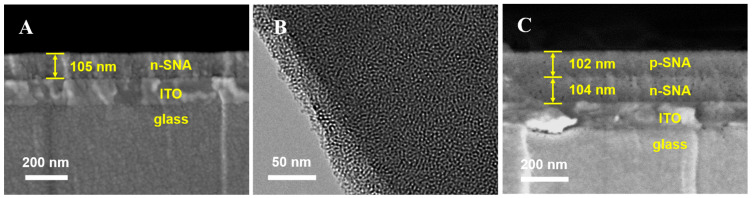
(**A**) SEM image of cross-sectional sample of n-SNA/ITO. (**B**) TEM image of n-SNA. (**C**) SEM image of cross-sectional sample of bp-SNA/ITO.

The morphology and structure of bp-SNA were characterized using SEM and TEM. Cross-sectional samples were obtained by cleaving the back of the n-SNA/ITO electrode using a blade, and SEM images clearly displayed its three-layer structure, including the glass substrate, ITO conductive layer, and the n-SNA layer (~105 nm), with well-defined interfaces (Figure 2A). TEM analysis revealed that n-SNA exhibited a highly ordered hexagonal mesoporous array with nanochannel diameters uniformly distributed between 2 and 3 nm (Figure 2B). After the further growth of the p-SNA layer, the cross-sectional SEM image displayed a four-layer structure, with the added p-SNA layer having a thickness of 102 nm, forming a symmetric bilayer system with the n-SNA layer (Figure 2C). This result confirmed that a bilayered structure with bipolar electrostatic nanocages was successfully constructed through the layered growth strategy.

**Figure 3:**
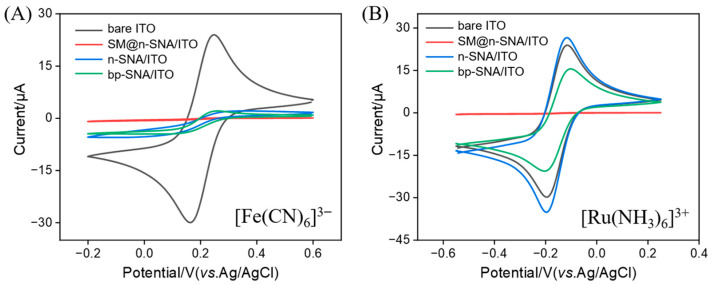
CV curves measured in different electrodes in presence of redox probe, including (**A**) Fe(CN)_6_^3−^ or (**B**) Ru(NH_3_)_6_^3+^.

The successful preparation of the bp-SNA/ITO electrode was validated through cyclic voltammetry analysis (Figure 3). The bare ITO electrode showed distinct redox peaks for the Fe(CN)_6_^3−^ probe. After the mesoporous nanochannels of the n-SNA were closed by the surfactant micelles (SM@n-SNA/ITO), the Faradaic current response almost disappeared, confirming that the nanochannel film had a defect-free and intact structure. For the n-SNA/ITO electrode, the peak current of Fe(CN)_6_^3−^ significantly decreased, which was attributed to the electrostatic repulsion between the negatively charged n-SNA layer and the same charge probe, significantly hindering the mass transport of the probe to the electrode surface. After the growth of the positively charged p-SNA layer on the n-SNA to form the bp-SNA/ITO, there was no significant change in the current response for Fe(CN)_6_^3−^, further confirming the dominant role of the inner n-SNA layer in repelling the negatively charged probe.

When the positively charged probe Ru(NH_3_)_6_^3+^ was selected, the peak current of the n-SNA/ITO was much higher than that of the bare ITO, indicating that the negatively charged n-SNA layer significantly enhanced the probe enrichment efficiency through electrostatic attraction. However, the current response of the bp-SNA/ITO was slightly lower than that of the n-SNA/ITO, which was attributed to the dynamic equilibrium formed between the positive electrostatic repulsion of the outer p-SNA layer and the electrostatic attraction of the inner n-SNA layer. This charge-selective transport phenomenon strongly confirmed the bipolar SNA structure and provided a basis for the stable confinement of the positively charged ECL emitter.

### 3.2. Stability of Ru(bpy)_3_^2+^ Enriched in bp-SNA

**Figure 4:**
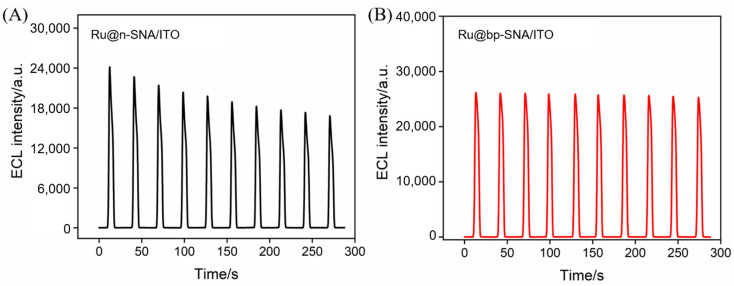
Continuously monitored ECL signal obtained on (**A**) Ru@n-VMSF/ITO or (**B**) Ru@bp-VMSF/ITO electrodes in TPA (3 mM).

The stabilizing effect of the bipolar nanocages on the ECL emitter was evaluated by measuring the ECL signal (Figure 4). The ECL signal intensity of the monolayer n-SNA/ITO electrode showed a significant decreasing trend as the scanning time increased (Figure 4A). After 10 consecutive measurements, the signal retention rate was less than 72%, indicating that the negatively charged nanochannels, relying solely on electrostatic attraction, could not effectively prevent the continuous loss of Ru(bpy)_3_^2+^ due to diffusion. In contrast, the bp-SNA/ITO electrode maintained 98.7% of its initial ECL signal after 10 consecutive measurements (Figure 4B), fully confirming that the cooperative effect of the bipolar electrostatic field could ensure the long-term stable immobilization of the probe. In addition, the measured ECL signal was higher than that obtained on the n-SNA/ITO electrode. This significant enhancement in stability was attributed to the electrostatic regulation of bipolar nanocages. In particular, the negatively charged inner n-SNA nanochannels effectively anchored the positively charged Ru(bpy)_3_^2+^ through electrostatic attraction, while the positively charged outer p-SNA nanochannels formed an electrostatic barrier that prevented the probe from diffusing into the solution phase. This stabilization strategy of the ECL emitter demonstrated several technical advantages. First, physical confinement was used as an alternative to the traditional chemical bonding method, avoiding the complex probe modification process. Second, the ion selectivity of the nanochannels significantly reduced the number of emitters required, with the ECL emitter concentration set at 10 μM, which is far lower than the conventional 100 μM used. This probe stabilization method, based on the bipolar nanoelectrostatic confinement effect, provides a new approach for the development of highly stable, solid-state ECL biosensors.

### 3.3. Interface Characteristics and ECL Signal on the Fabrication of Enzyme Electrodes

**Figure 5:**
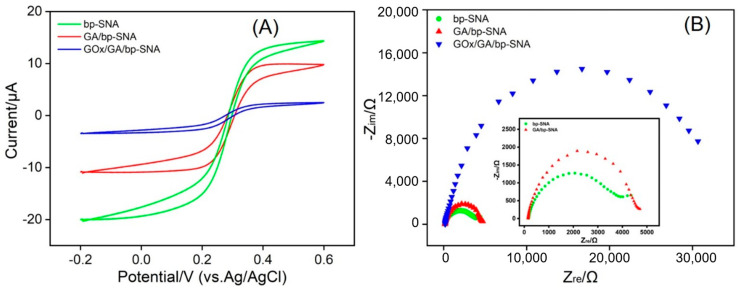
The CV (**A**) and EIS (**B**) spectra of bp-SNA, GA/bp-SNA, and GOx/GA/bp-SNA in a 0.1 M KCl solution containing Fe(CN)_6_^3−^/^4−^ (2.5 mM). The scan rate in panel A was 100 mV/s.

The interface electron transfer properties of the electrodes during the stepwise modification process for enzyme electrode construction were studied using CV and EIS (Figure 5). Fe(CN)_6_^3−^/^4−^ was used as the redox probe. The results show that the original bp-SNA/ITO electrode exhibited a typical reversible redox peak (Figure 5A). After crosslinking with GA, the peak current of the GA/bp-SNA/ITO electrode decreased, indicating that the aldehyde groups had partially hindered the diffusion of the probe, thus confirming the successful anchoring of GA. The further immobilization of GOx led to a significant decrease in the peak current of the GOx/GA/bp-SNA/ITO electrode. This reduction was attributed to the steric hindrance of the enzyme protein molecules and their non-conductive properties, which blocked the electron transfer path. EIS analysis further revealed the changes in the charge transfer resistance (*R*ct) of different electrode interfaces (Figure 5B). Consistent with the CV results, the *R*ct of the electrode after GA modification, particularly after GOx immobilization, significantly increased. This was due to the insulating nature of the fixed GOx molecules on the electrode surface, which reduced the migration of co-reactants and electrolyte ions at the interface. Additionally, three-dimensional protein conformation created a barrier for the diffusion of Fe(CN)_6_^3−^/^4−^.

**Figure 6:**
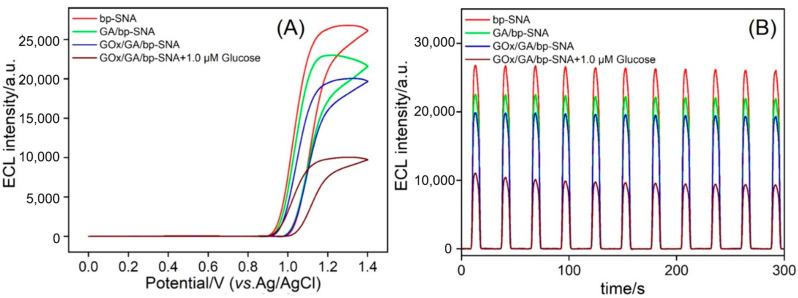
(**A**) *I*_ECL_–potential or (**B**) *I*_ECL_–time curves of bp-SNA, GA/bp-SNA, GOx/GA/bp-SNA in 3 mM TPA (pH = 7.4 0.01 M PBS). The scan rate in (**A**) was 100 mV/s.

The feasibility of the sensor construction process was further evaluated by measuring the ECL signal of the electrodes (Figure 6). In a buffer system containing TPA, the original bp-SNA/ITO electrode exhibited a stable ECL signal. After GA crosslinking, the ECL intensity decreased, which was attributed to the crosslinking between the aldehyde groups and the amine groups. Upon immobilizing GOx, the ECL signal further dropped sharply. This was due to the steric shielding effect of the GOx molecules formed on the electrode surface, which significantly inhibited the diffusion of TPA into the nanocavities. In addition, the insulating properties of the enzyme proteins increased the electrode interface’s resistance. The introduction of glucose resulted in a further reduction in the ECL signal, primarily due to the enzymatic oxidation of glucose by GOx, which produced H_2_O_2_ as a byproduct. In the Ru(bpy)_3_^2+^/TPA system, the presence of H_2_O_2_ disrupts this ECL process by acting as a quencher [65,66].

The trend of ECL intensity change was consistent with the results from CV and EIS characterizations. In addition, the relative standard deviation (RSD) of the ECL signal for each electrode during the 300 s continuous scanning in the enzyme electrode construction process was below 5%, confirming that the electrostatic confinement effect of the nanochannels effectively maintained the stability of the immobilized emitter during the electrode modification process.

### 3.4. ECL Detection of Glucose and Detection Selectivity

**Figure 7:**
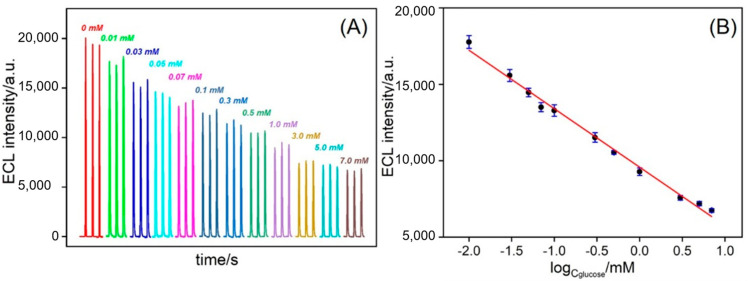
(**A**) The ECL response of GOx/Ru/GA/bp-SNA/ITO to the continuous addition of glucose in 0.01 M PBS containing TPA (3 mM). (**B**) The linear calibration curve for glucose detection. The error bars represent the standard deviations of three measurements.

The constructed enzyme sensor was used to detect a series of glucose concentrations. Figure 7A shows the ECL curves obtained at different glucose concentrations. It was observed that the ECL intensity decreased as the glucose concentration increased, exhibiting an inverse-proportional relationship. This was because a higher glucose concentration in the solution led to more H_2_O_2_ generated by the enzymatic reaction, resulting in a stronger quenching effect. The ECL intensity (*I*) was fitted with a glucose concentration (*C*_glucose_) to obtain a standard linear response for glucose detection. A good linear relationship was observed between glucose concentrations ranging from 10 μM to 7.0 mM (Figure 7B), with the linear regression equation being *I*_ECL_ = −3827.7log*C*_glucose_ + 9576.9 (*R*^2^ = 0.992). The limit of detection (LOD), calculated based on a signal-to-noise ratio of 3 (S/N = 3), was 1.0 μM. The limit of quantification (LOQ) based on a signal-to-noise ratio of 10 (S/N = 10) was 3.3 μM.

**Figure 8:**
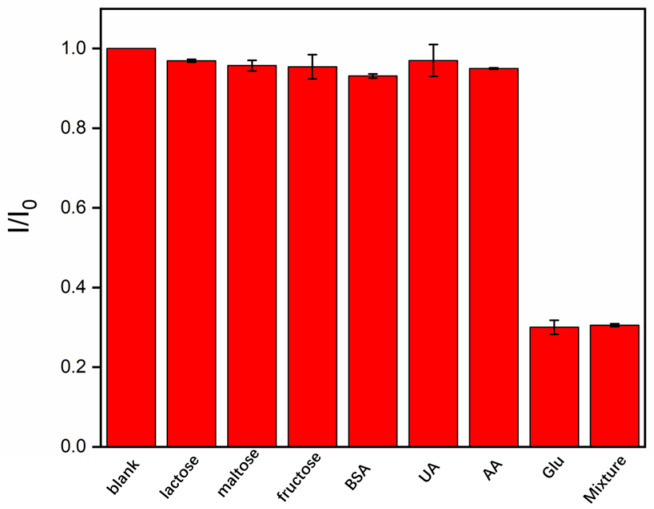
The ECL signal ratio (*I*/*I*_0_) of GOx/Ru/GA/bp-SNA/ITO in a solution containing 3 mM glucose and TPA (3 mM) upon the addition of different interferents (150 mM). Here, *I*_0_ and *I* represent the ECL intensity measured on the electrode before and after the addition of the substance, respectively.

Specificity remains a critical challenge in the field of glucose sensing, particularly due to the difficulty of discriminating glucose from structurally similar molecules, such as fructose, galactose, and even ascorbic acid (AA), which can compromise sensor accuracy and reliability. To evaluate cross-reactivity, the sensor response to structurally related sugar, including lactose, maltose, and fructose, was investigated. As shown in Figure 8, even at concentrations 50-fold higher than that of glucose, these substances induced no significant change in the ECL signal. Furthermore, the influence of common biological interferents such as AA, dopamine (DA), and the large protein (bovine serum albumin, BSA) was examined. The results demonstrated negligible signal change, indicating excellent specificity. This high specificity can be attributed to three key factors: (1) the intrinsic stereoselectivity of the enzyme toward glucose; (2) the size-exclusion effect of the nanochannels (2–3 nm diameter), which effectively prevented the entry of large molecular interferents; and (3) the bipolar electrostatic environment within the nanochannels, which suppressed the non-specific adsorption of charged species.

### 3.5. Analysis of Real Sample

The accuracy of the method was verified through additional recovery experiments. The experimental results, as shown in Table 1, demonstrate that both the GOx/Ru/GA/bp-SNA/ITO electrodes exhibited good recovery rates (96.2–102.5%) and low RSD values (<2.0%) for glucose detection in diluted FSA. These results indicate that the method possessed good accuracy.

## 4. Conclusions

In this work, a bipolar nanostructure (bp-SNA) was constructed on the surface of the ITO electrode, and the electrostatic confinement of the Ru(bpy)_3_^2+^ emitter was achieved using a charge gradient field. The negatively charged inner n-SNA layer generated a strong enrichment effect, while the positively charged outer p-SNA layer formed a dynamic barrier, stabilizing the ECL emitter within the electrode surface. Additionally, enzyme molecules were covalently immobilized through a Schiff base reaction mediated by glutaraldehyde. Finally, based on the quenching effect of H_2_O_2_ generated in situ via an enzyme-catalyzed reaction, a glucose detection method was established, enabling the selective and sensitive detection of glucose. This strategy significantly improved the stability of the immobilized emitter, detection selectivity, and anti-interference ability, offering the potential for the development of solid-state biosensor platforms for biomarker detection. The solid-phase ECL platform constructed in this study holds promise as a universal metabolite detection platform. For example, by replacing the enzymes (such as urease, cholesterol oxidase, etc.) immobilized on the surface of the SNA, this method can also be applied to the detection of metabolites like uric acid or cholesterol. Additionally, the use of cost-effective and readily available ITO electrodes as the supporting electrode, along with the simple and low-cost preparation method for bp-SNA, offers potential for the development of disposable electrodes.

## Figures and Tables

**Table 1:** The detection of glucose using the fabricated solid ECL enzyme sensor in FBS.

Sample	Added (mM)	Detected (mM)	RSD (%, n = 3)	Recovery (%)
fetal bovine serum ^a^	0.10	0.10	0.7	100.0
	0.50	0.48	0.3	96.2
	1.00	1.02	2.0	102.5

^a^ Fetal bovine serum was diluted 50 times using the electrolyte.

## Data Availability

The data presented in this study are available upon request from the corresponding author.
